# Conservative Management of Ureteral Injury Caused by a Lumbar Osteophyte

**DOI:** 10.1089/cren.2016.0126

**Published:** 2016-12-01

**Authors:** Max Fuller, Michael Brekhus

**Affiliations:** ^1^ Sanford School of Medicine, University of South Dakota, Rapid City, South Dakota.; ^2^ Department of Surgery, Sanford School of Medicine, University of South Dakota, Rapid City, South Dakota.

**Keywords:** lumbar osteophyte, ureter injury, disk space extravasation

## Abstract

***Background:*** Osteophytes are bony outgrowths commonly found on lumbar vertebrae. They rarely produce complications with the most common complication being nerve entrapment, but rarer complications including aorta or inferior vena cava rupture, superior mesenteric artery syndrome, compression of the iliopsoas muscle, and cerebrospinal fluid leaks have been described. Rare cases affecting the ureter resulting in ureteral colic or extravasation of urine have been described.

***Case Presentation:*** We describe a case in which a lumbar osteophyte bridging the L4 and L5 disks was encircling the ureter and minor trauma caused a ureteral injury, resulting in urine extravasation into the L4 and L5 disks space and the retroperitoneum. Owing to the comorbidities of this patient, this case was treated conservatively with stenting and the patient has suffered no further complications.

***Conclusion:*** This is a rare complication of a lumbar osteophyte but should be considered as a potential cause of ureter injury. Treatment should be individualized by patient preference and comorbidities, as some patients would elect to pursue more aggressive therapy whereas others would incline for conservative measures.

## Introduction and Background

Osteophytes extending from lumbar vertebrae are a common incidental radiologic finding especially with increasing age. Fortunately, complications from osteophytes are rare with nerve compression resulting in sciatica being the most common. Some of the more rare complications that have been reported include aorta or inferior vena cava (IVC) rupture, superior mesenteric artery syndrome, compression of the iliopsoas muscle, and cerebrospinal fluid leaks.^1^ There have been rare complications of osteophytes affecting the ureters, causing ureteral colic because of obstruction and urine extravasation of urine, and these cases have been repaired surgically with end to end anastomosis.^2,3^ Ureteral injuries resulting in extravasation are exceedingly rare with most common injuries caused iatrogenically during gynecologic or abdominal surgery or from penetrating trauma. These are generally repaired surgically, either open or laproscopically. This case describes a rare cause of ureteral injury secondary to a lumbar osteophyte from the L4 and L5 vertebrae encircling the ureter that resulted in extravasation of urine into the retroperitoneum and L4 and L5 disks space.

## Presentation of Case

**FIG. 1.**
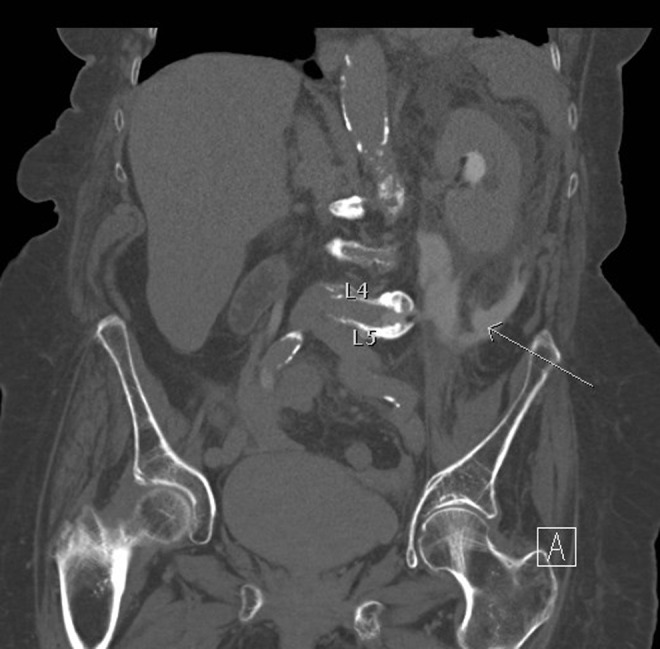
CT scan with contrast illustrating vertebral osteophytes extending from L4 and L5 vertebrae encircling ureter. Contrast is seen extravasating into the L4 and L5 disks space and the infrarenal space. *Arrow* demonstrates contrast extravasating into the L4 and L5 disk space and infrarenal space. UPJ = ureteropelvic junction.

We report an 88-year-old female who presented to the local emergency room after a backward fall. She had a history chronic obstructive pulmonary disease, hypothyroidism, and hypertension and mild congestive heart failure. She was also a former smoker but had been relatively stable in managing her chronic conditions. CTs of the head and C-spine were performed and were negative for acute changes. She also had nausea and vomiting after the fall, which persisted despite antiemetics. A white blood cell (WBC) count of 14.4 with 87% neutrophils was also noted initially, but she did not show any signs of infection. Other laboratories were unremarkable, including a blood urea nitrogen (BUN) of 30 and a creatinine of 1.0. Owing to the nausea and vomiting, she was admitted overnight for observation. The next day, she continued to have nausea and vomiting and also complained of left flank pain radiating to the groin. A CT of the abdomen and pelvis without IV contrast was performed to look for an etiology of her symptoms. The scan showed apparent pyelonephritis, hydronephrosis, and obstruction of the upper 1/3 of the ureter but no stones. Vertebral osteophytes extending laterally from the L4 and L5 vertebrae were discovered and these appeared to be encircling the left ureter. The decision was then made to transfer her to a higher level of care for urologic services. After transfer, a CT urogram showed extravasation of the contrast into the L4 and L5 disks space and the infrarenal space, indicating ureteral injury approximately 5 cm below the UPJ (Fig. 1). Despite the ureter obstruction, her WBC count continued to decline to 11.7 and her BUN and creatinine upon transfer were 17 and 0.8, respectively. A urinalysis showed moderate occult blood, 0–4 red blood cells (RBCs), and 15–29 WBCs, but was negative for leukocyte esterase and nitrites. Urine culture eventually grew out *Enterobacter aerogenes* and she was treated with ciprofloxacin. Owing to the extravasation of contrast into the disk space, neurosurgery was also consulted for a potential infection of the disk space but she was not showing any signs of a significant infection, so it was believed that more aggressive treatment was not warranted. Removing the osteophyte that was causing these problems was also discussed, but this would have subjected her to an invasive surgery that she would likely have not tolerated well.

**FIG. 2.**
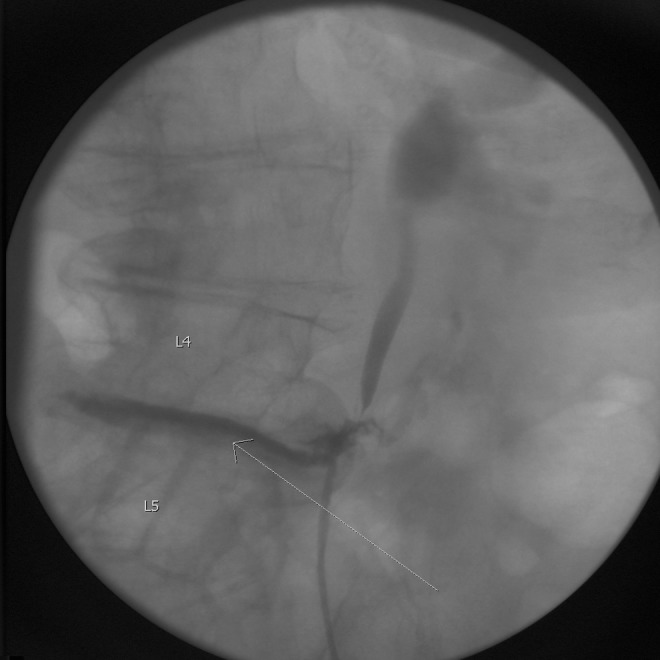
Retrograde pyelography showing contrast extravasation into L4 and L5 disks space. *Arrow* demonstrates contrast extravasation into the L4 and L5 disk space.

**FIG. 3.**
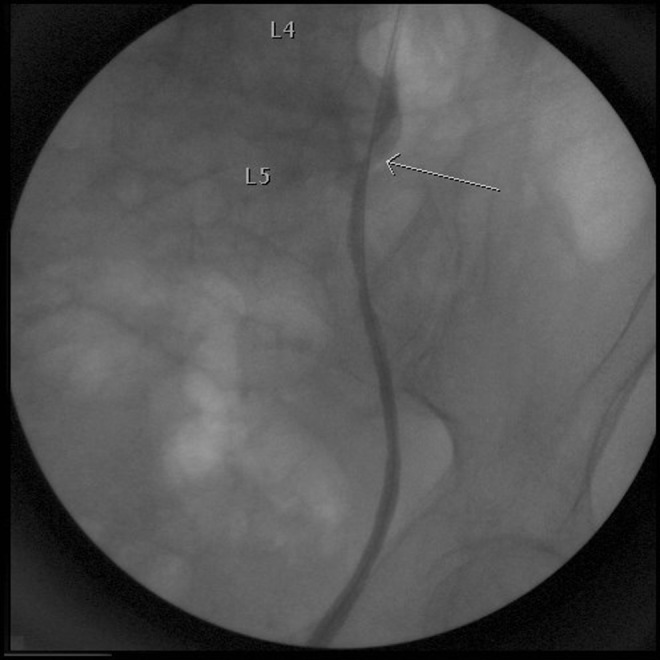
Retrograde pyelography showing ureteral stricture that has formed since initial intervention. *Arrow* demonstrates area of ureteral narrowing and stricture formation.

The decision was made to proceed with cystoscopy and retrograde pyelogram to better identify the ureter injury. No abnormalities were noted in the bladder but there was no urine effluxing from the left ureteral orifice. Retrograde pyelogram showed extravasation of contrast in the location of the L4 and L5 osteophytes and was again noted to fill the disk space between L4 and L5 (Fig. 2). With great care, a wire was advanced into the proximal ureter and a 6F, Double-J ureteral stent was placed. A Foley catheter was placed in the bladder as well. After the hospitalization, the patient and her family felt that risk of operative intervention for reconstruction was too significant because of her age and other comorbidites and she elected to manage the situation with the ureteral stent. During follow-up, retrograde pyelography has confirmed no persistent extravasation, but the region of the ureteral injury has developed a stricture with poor drainage of contrast through the narrowed area (Fig. 3). Despite this stricture, she has not developed any further problems related to her injury and she continues to be followed with serial stent exchanges.

## Discussion and Literature Review

This is a rare case of ureteral injury but because of the prevalence of lumbar osteophytes, entrapment by an osteophyte may be a cause of ureteral obstruction in certain presentations. Our patient elected for conservative management and has done reasonably well for more than 2 years with serial stent exchanges. More aggressive treatment would likely require a multidisciplinary operation with urology, neurosurgery, or orthopedics. Surgical repair of the ureteral injury and removal of the osteophyte would have likely required anterior spinal access. This approach requires mobilization of the aorta, IVC, or iliac vessels depending on which level of the spine is being accessed. This procedure has a large range of potential complications with the most serious being injury to the vasculature or spinal nerve roots.^4^ Lumbar osteophytes are most commonly removed if causing sciatica, but this is usually done by a much less invasive posterior approach. In this case, stenting has maintained a patent ureter without any evidence of worsening renal function or pain. A more aggressive therapy may be warranted in younger patients but stenting has proven to manage this injury quite well. Despite the stricture that formed, we feel that our patient achieved a good outcome with the conservative approach and we would not have changed the initial management decision.

## Conclusion

Ureteral injury is a rare complication of lumbar osteophytes but should be considered as a potential cause of ureteral obstruction and injury. Both surgical management with primary repair or management with stenting of the affected segment can be considered for treatment.

## Abbreviations Used

BUN: blood urea nitrogen
CT: computed tomography
IVC: inferior vena cava
WBC: white blood cell
